# Periodical Literature

**Published:** 1873-11

**Authors:** 


Periodical Literature.—St. Nicholas is the title of a new magazine for Girls and Boys, the first number of which has been issued by Scribner & Co., New York. The magazine is under the Editorial management of Mrs. Mary Mapes Dodge, well known as the author of the Irvington Stories, Hans Brinker, etc. The number before us gives promise of a rich treat for its young readers and the contents evince an admirable appreciation of their wants. If all of the many books and magazines designed for children were so well adapted to their wants less hesitation would be manifested by parents and teachers in welcoming this class of literature. Although St. Nicholas seems to have arrived this year in a new form and a little in advance of his usual time, we think he will be none the less welcome. The subscription price of the magazine has been fixed at $3.00 a year.

—The publishers of Wood's Household Magazine offer in connection with their publication as a premium to subscribers a large oil chromo entitled The Yosemite. We have not seen the chromo, but from the small engraving of it which accompanies the magazine judge it to be quite fine, and dou t not that it is equal to all that is claimed for i. The magazine and chromo together are $1 50.

